# Association of diabetes with atrial fibrillation types: a systematic review and meta-analysis

**DOI:** 10.1186/s12933-021-01423-2

**Published:** 2021-12-07

**Authors:** Fadi Alijla, Chepkoech Buttia, Tobias Reichlin, Salman Razvi, Beatrice Minder, Matthias Wilhelm, Taulant Muka, Oscar H. Franco, Arjola Bano

**Affiliations:** 1 Institute of Social and Preventive Medicine (ISPM), University of Bern, Mittelstrasse 43, 3012 Bern, Switzerland; 2 Department of Cardiology, Inselspital, Bern University Hospital, University of Bern, Bern, Switzerland; 3 Department of Endocrinology, Gateshead Health NHS Foundation Trust, Gateshead, UK; 4 Translational and Clinical Research Institute, Newcastle University, Newcastle upon Tyne, UK; 5 Public Health and Primary Care Library, University Library of Bern, University of Bern, Bern, Switzerland

**Keywords:** Diabetes mellitus, Paroxysmal, Non-paroxysmal, Persistent, Permanent, Atrial fibrillation

## Abstract

**Background:**

Atrial fibrillation (AF) is a common arrhythmia classified as paroxysmal and non-paroxysmal. Non-paroxysmal AF is associated with an increased risk of complications. Diabetes contributes to AF initiation, yet its role in AF maintenance is unclear. We conducted a systematic review and meta-analysis to summarize the evidence regarding the association of diabetes with AF types.

**Methods:**

We searched 5 databases for observational studies investigating the association of diabetes with the likelihood of an AF type (vs another type) in humans. Study quality was evaluated using the Newcastle–Ottawa Scale. Studies classifying AF types as paroxysmal (reference) and non-paroxysmal were pooled in a meta-analysis using random effects models.

**Results:**

Of 1997 articles we identified, 20 were included in our systematic review. The population sample size ranged from 64 to 9816 participants with mean age ranging from 40 to 75 years and percentage of women from 24.8 to 100%. The quality of studies varied from poor (60%) to fair (5%) to good (35%). In the systematic review, 8 studies among patients with AF investigated the cross-sectional association of diabetes with non-paroxysmal AF (vs paroxysmal) of which 6 showed a positive association and 2 showed no association. Fourteen studies investigated the longitudinal association of diabetes with “more sustained” AF types (vs “less sustained”) of which 2 showed a positive association and 12 showed no association. In the meta-analysis of cross-sectional studies, patients with AF and diabetes were 1.31-times more likely to have non-paroxysmal AF than those without diabetes [8 studies; pooled OR (95% CI), 1.31 (1.13–1.51), I^2^ = 82.6%]. The meta-analysis of longitudinal studies showed that for patients with paroxysmal AF, diabetes is associated with 1.32-times increased likelihood of progression to non-paroxysmal AF [five studies; pooled OR (95% CI), 1.32 (1.07–1.62); I^2^ = 0%].

**Conclusions:**

Our findings suggest that diabetes is associated with an increased likelihood of non-paroxysmal AF rather than paroxysmal AF. However, further high quality studies are needed to replicate these findings, adjust for potential confounders, elucidate mechanisms linking diabetes to non-paroxysmal AF, and assess the impact of antidiabetic medications on AF types. These strategies could eventually help decrease the risk of non-paroxysmal AF among patients with diabetes.

**Supplementary Information:**

The online version contains supplementary material available at 10.1186/s12933-021-01423-2.

## Introduction

Atrial fibrillation (AF) is the most common arrhythmia, and it is associated with high risk of morbidity and mortality [1–4]. The incidence and prevalence of AF are increasing worldwide [5]. According to recent guidelines, AF is classified into the following types: first diagnosed, paroxysmal, persistent, long-standing persistent, and permanent AF (Additional file 1: Table S1) [6]. While paroxysmal AF terminates within 7 days of onset, non-paroxysmal AF (including persistent, long-standing persistent, and permanent AF) lasts longer [6]. Patients who develop non-paroxysmal AF have a higher risk of adverse events (including all-cause mortality, thromboembolism, and hospitalization) compared to those with paroxysmal AF [7, 8]. Therefore, the identification of modifiable risk factors for non-paroxysmal AF is important to improve pathophysiological understandings about AF development and to improve clinical management and prognosis for patients with AF by avoiding AF progression to non-paroxysmal AF.

Diabetes mellitus represents a major risk factor for AF [9]. Patients with diabetes have a 34% greater risk of AF compared to those without diabetes [10]. Several mechanisms, such as electrical, structural, and autonomic atrial remodelling, can explain the role of diabetes in the initiation of AF [11]. However, the impact of diabetes on the maintenance of sustained AF in non-paroxysmal AF remains unelucidated. To date, epidemiological studies have investigated the association of diabetes with different classifications of AF types [12–15]. Various forms of “more sustained AF” (i.e., non-paroxysmal AF, permanent AF, AF progression of paroxysmal to non-paroxysmal AF, and persistent to permanent AF) have been compared with forms of “less sustained AF.” However, results across studies have been inconsistent. For example, one study reported that among patients diagnosed with AF, those with and without diabetes have significant differences in AF types [13], while another study found no differences [16]. A study including patients with paroxysmal AF reported an association between diabetes and the development of permanent AF [14], yet another study found no association [12]. These inconsistencies may also arise from differences in definitions of AF types, differences in characteristics of participants, or small study sample sizes. Overall, it is unclear whether patients with and without diabetes have differences in AF types.

We therefore performed a systematic review, which aimed to summarize evidence regarding the association of diabetes with the likelihood of having a certain AF type, rather than another AF type. In order to provide insights concerning the role of diabetes on AF’s pathophysiology, we also performed a meta-analysis of diabetes’ association with the likelihood of having non-paroxysmal AF rather than paroxysmal AF.

## Methods

### Data sources and search strategy

We conducted our systematic review following a recently published guide about performing systematic reviews and meta-analyses [17]. The guide was based on the Preferred Reporting Items for Systematic Review and Meta-analyses recommendations [18]. We searched Embase, Medline Ovid, Cochrane Central, Web of Science Core Collection, and Google Scholar from inception until 1 October 2021 [18]. To identify relevant articles, we combined (a) diabetes-related terms with (b) AF-related terms, such as “atrial,” “fibrillation,” “paroxysmal,” “persistent,” “permanent,” and (c) article type. We excluded conference abstracts, letters to the editor, and editorials. (Additional file 1: Appendix A). We performed our search with the assistance of an experienced information specialist. We used EndNote to manage references.

### Study selection

Eligibility criteria for included studies required (1) observational studies, including cross-sectional, prospective, case–control, nested case–control, or nested case-cohort studies of humans that (2) investigated the association of diabetes with the likelihood of having a certain AF type rather than another AF type and (3) provided information about effect estimates (e.g., risk ratios, hazard ratios [HRs], and odds ratios [ORs]) with 95% confidence intervals [95% CIs] or p-values). We excluded animal studies, reviews, meta-analyses, conference abstracts, conference proceedings, poster presentations, case-series, and letters to the editor. We did not restrict publication year or language.

Two reviewers independently screened eligible citations by title and abstract. Furthermore, these two reviewers independently evaluated full texts of eligible articles. In cases of disagreement between reviewers, decisions were either made by consensus or in consultation with a third reviewer. We hand searched the reference lists of included articles to identify additional studies.

### Data extraction

Two authors independently extracted the following data from each study: first author`s last name, year of publication, country where the study was conducted, study population, number of participants at baseline, mean age, percentage of women, diabetes assessment, adjustments for potential confounders, outcome, follow-up time, and risk estimates with 95% CIs.

### Quality assessment

Two authors independently performed quality assessment of the included studies using the Newcastle–Ottawa Scale (NOS) [19]. NOS rates study quality from 0 to 9 stars according to three domains: selection, comparability, and outcome assessment. Based on thresholds for converting NOS scores into Agency for Healthcare Research and Quality standards, we categorized study quality as (I) good (selection: 3–4 stars; comparability: 1–2; outcome assessment: 2–3); (II) fair (selection: 2; comparability: 1–2; outcome assessment: 2–3); or (III) poor (selection: 0–1; comparability: 0; outcome assessment: 0–1) (Additional file 1: Appendix B) [19, 20].

### Statistical analyses

We included eligible studies that classified AF types into paroxysmal (reference) and non-paroxysmal AF in the meta-analysis. When applicable, ORs and 95% CIs were calculated using 2 by 2 tables. Effect estimates on the association of diabetes with the likelihood of non-paroxysmal AF (vs paroxysmal AF) were pooled using random effects models as described by DerSimonian and Laird; and forest plots were constructed [21]. We assessed heterogeneity using the I^2^ statistic; we considered I^2^ ≤ 25% low; 25% < I^2^ < 75% moderate; and I^2^ ≥ 75% high. In order to test the robustness of our results, we performed several sensitivity analyses: (1) we recalculated estimates after removing studies one by one from the pooled analysis to evaluate the role of individual studies on the overall results; (2) we performed subgroup analyses based on follow-up time (i.e., ≤ 1 year vs > 1 year) in our meta-analysis of longitudinal studies; and (3) we also restricted our meta-analysis of longitudinal studies to studies that defined AF types in accordance with the guidelines [6]. We used funnel plots and Egger regression symmetry tests to evaluate the possibility of publication bias [22]. Statistical analyses were performed using Stata IC version 15.1 (StataCorp LLC, Texas, USA).

## Results

### Literature search

After excluding duplicates, we identified 1,997 relevant citations. We further screened studies by title and abstract, identifying 33 potentially relevant articles. After examining the article full texts, we identified 17 unique eligible articles. Our reference search yielded 3 additional articles, resulting in a total of 20 articles that we included in our systematic review. We present the results of our search strategy in Additional file 1: Fig. S1.

### Characteristics of included studies

The population sample size ranged from 64 to 9816 participants. The mean age ranged from 40 to 75 years. The percentage of women ranged from 24.8 to 100%. We present the general characteristics of the 20 eligible articles in Tables 1 and 2 and Additional file 1: Table S2. Of the 20 included studies, 5 were conducted in the United States [12, 23–26], 8 in Europe [7, 13–16, 27–29], 3 in Japan [30–32], 1 in Canada [33], 1 in Australia [34], and 2 across several continents [35, 36]. Four studies diagnosed diabetes based on medical files and/or use of antidiabetic treatment [12, 23, 24, 32] (Tables 1 and 2). Sixteen studies did not provide information about assessment of diabetes [7, 13–16, 25–31, 33–37] (Tables 1 and 2). We provide detailed information about the studies’ recruitment settings and periods, inclusion and exclusion criteria, AF monitoring, and AF definitions across studies in Additional file 1: Tables S2, S3 and S4.

**Table 1:** Description of studies investigating the cross-sectional association of DM with AF types

	First author, year (Reference)	Country	N	Population	Mean age (years)	Women %	DM assessment	Adjustment for potential confounders	Outcome (Ref)	Result
1*†	Nabauer, 2009	Germany	7907	Paroxysmal, persistent, and permanent AF	68.4 ± 11.0	38.8	NR	-	Non-paroxysmal AF [i.e., persistent or permanent] (Ref: paroxysmal AF)	**uOR (CI), 1.74 (1.54–1.96)**
2*†	Chiang, 2012	26 countries in Europe, Asia, Africa, and South America	9816	Paroxysmal, persistent, and permanent AF	68.3	44.2	NR	-	Non-paroxysmal AF [i.e., persistent or permanent] (Ref: paroxysmal AF)	**uOR (CI), 1.20 (1.07–1.34)**
3*†	Boriani, 2016	9 European countries	1815	Paroxysmal, persistant, and permanent AF	40.15	39.8	NR	-	Non-paroxysmal AF [i.e., persistent or permanent] (Ref: paroxysmal AF)	**uOR (CI), 1.40 (1.12–1.82)**
4*†	Echouffo-Tcheugui, 2017	United States	6575	Paroxysmal, persistent, and permanent AF	75	42.6	Previous medical history or new DM diagnosis during enrollment visit	–	Non-paroxysmal AF [i.e., persistent or permanent] (Ref: paroxysmal AF)	**uOR (CI), 1.18 (1.08–1.29)**
5*†	Fumagalli, 2018	9 European countries	2337	Paroxysmal, persistent, long-standing persistent and permanent AF	69	40.3	NR	–	Non-paroxysmal AF [persistent, long-standing persistent or permanent] (Ref: paroxysmal AF)	**uOR (CI), 1.46 (1.17–1.82)**
6*	Ruperti 2018	Switzerland	476	Paroxysmal, persistent, and permanent AF	68	67.5	NR	–	Recent non-paroxysmal AF [i.e., persistent or permanent] (Ref: paroxysmal AF)	uOR (CI), 0.94 (0.53–1.67)
								Age, sex, BMI, HTN, heart rate, CAD, HF, LVH education, history of hyperthyroidism, smoking, physical activity, alcohol		aOR (CI), 0.79 (0.40–1.54)
7*†	Schnabel, 2018	7 European countries	3210	Paroxysmal, persistent, and permanent AF	72	39.9	NR	–	Non-paroxysmal AF [i.e., persistent or permanent] (Ref: paroxysmal AF)	uOR (CI), 1.03 (0.87–1.21)
8*	Bhat, 2021	Australia	665	Paroxysmal, persistent, and permanent AF	66.8 ± 13.5	47	NR	–	Non-paroxysmal AF [i.e., persistent or permanent] (Ref: paroxysmal AF)	**uOR (CI), 1.55 (1.09–2.20)**
								Age, HF, ischemic heart disease, anticoagulation, left ventricular ejection fraction, right atrial area, average E/e`, left atrial emptying fraction		aOR (CI), 1.42 (0.80–2.54)

*The studies investigated the association of diabetes with non-paroxysmal AF (compared to paroxysmal AF)

^†^Unadjusted odds ratios for developing non-paroxysmal AF compared to paroxysmal AF were calculated manually

*DM* diabetes mellitus, *AF* atrial fibrillation, *NR* not reported, *uOR* unadjusted odds ratio, *BMI* body mass index, *HTN* hypertension, *CAD* coronary artery disease, *HF* heart failure, *LVH* left ventricular hypertrophy, *aOR* adjusted odds ratio, *CI* 95% confidence interval, *Ref* reference

**Table 2:** Description of studies investigating the longitudinal association of DM with AF types

	First author, year (Reference)	Country	N	Population	Mean age (years)	Females (%)	DM assessment	Adjustment for potential confounders	Outcome (Ref)	Follow-up time	Result
A. Studies providing hazard ratios (Method: Cox regression)											
1	Tsang, 2007	United States	3248	Patients with first episode of paroxysmal AF	71 ± 15	46	DM medical diagnosis or treatment with antidiabetic medications	Age, sex	Permanent AF (Ref: no AF recurrence, recurrent paroxysmal AF, recurrent persistent AF)	Median (IQR), 5.1 (1.2–9.4) y	aHR (CI), 1.17 (0.94–1.47)
2	Pappone, 2008	Italy	106	Patients with first episode of paroxysmal AF	57.5	35.8	NR	Age, HF	Permanent AF (Ref: no AF recurrence, recurrent paroxysmal AF, recurrent persistent AF)	Maximum, 5 y	**aHR (CI), 17.37 (3.75–80.43)**
3	Kawara, 2010	Japan	64	Paroxysmal or persistent AF	61 ± 10	29.6	NR	Age, sex, HTN, organic heart disease, HF, severity of symptoms	Permanent AF (Ref: non-permanent AF)	Median (IQR), 4.9 (2.4–8.9) y	aHR (CI), 3.13 (0.46–21.2)
4	Thacker, 2013	United States	1385	Patients whose initial AF episode terminated within 6 months	69.2	48.7	DM medical diagnosis, and current use of insulin or oral hypoglycemic medication	Age, sex	Permanent AF (Ref: non-permanent AF)	Mean (range), 7 (5–8) y	Model 1 aHR (CI), 0.99 (0.72–1.36)
								Age, sex, BMI, HTN, SBP, DBP			Model 2 aHR (CI), 0.93 (0.67–1.29)
								Age, sex, BMI, HTN, SBP, DBP, CHD, valvular heart disease, HF, stroke			Model 3 aHR (CI), 0.94 (0.67–1.32)
5	Senoo, 2014	Japan	1176	Paroxysmal AF	61.4 ± 13.1	25.6	NR	-	Recurrent AF (Ref: non-recurrent AF)	Mean (sd), 3.3 ± 2.5 y	uHR (CI), 1.47 (0.89–2.44)
6*	Sandhu, 2014	United States	1039	Patients who developed AF in a cohort free of AF	58.9	100	NR	Age, aspirin, vitamin E, beta‐carotene, BMI, HTN, cholesterol, alcohol, smoking, exercise	Non-paroxysmal AF [i.e., persistent or permanent] (Ref: paroxysmal AF)	Median (IQR), 16.4 (15.6–16.8) y**	Model 1 aHR (CI), 1.04 (0.72 to 1.41)***
								Age, MI, stroke, revascularization, HF			Model 2 aHR (CI), 1.06 (0.69–1.5)***
7	Blum, 2019	Switzerland	2869	Paroxysmal or persistent AF	70 ± 9	32.3	NR	Age, sex	AF Progression [i.e., paroxysmal to persistent or permanent, persistent to permanent] (Ref: paroxysmal or persistent AF)	Median (IQR), 3 (2–5) y	Model 1 aHR (CI), 1.14 (0.88–1.48)
								Age, sex, BMI, heart rate, SBP, coronary artery disease, HTN, stroke/TIA, HF, hyperthyroidism, history of renal failure, physical activity, smoking, history of pulmonary vein isolation, AF‐related symptoms, amiodarone			Model 2 aHR (CI), 0.92 (0.69–1.21)
B. Studies providing odds ratios (Method: Logistic regression)											
8*†	Sakamoto, 1995	Japan	137	Paroxysmal AF	64	24.8	Use of antidiabetic therapy	–	Chronic AF (Ref: paroxysmal AF)	Mean, 1 y	**uOR (CI), 2.52 (1.05–6.05)**
9*†	Kerr, 2005	Canada	757	Paroxysmal AF	64	38.3	NR	–	Chronic AF (Ref: paroxysmal AF)	8 (0–11) y	uOR (CI), 1.07 (0.60–1.92)
10*	Pillarisetti, 2009	United States	437	Paroxysmal AF	67.9 ± 13.4	43	NR	–	Non-paroxysmal AF [i.e., persistant or permanent] (Ref: paroxysmal AF)	Mean (sd), 4.7 ± 4.6 y	uOR (CI), 1.50 (0.90–2.60)
11*†	de Vos, 2010	35 European countries	1219	Paroxysmal AF and first detected AF in whom sinus rhythm restored spontaneously or after treatment during admission	64	43	NR	–	Non-paroxysmal AF [i.e., persistent or permanent] (Ref: paroxysmal AF)	Mean, 1 y	uOR (CI), 1.42 (0.94–2.14)
12*†	de Vos, 2012	21 countries in Europe, North and South America, Asia	2137	Paroxysmal and first-detected AF	65 ± 12	NR	NR	–	Non-paroxysmal AF [i.e., persistent or permanent] (Ref: paroxysmal AF)	Mean, 1 y	uOR (CI), 1.18 (0.86–1.63)
13	Echouffo-Tcheugui, 2017	United States	6575	Paroxysmal or persistent AF	75	42.6	Previous medical history or new DM diagnosis during enrollment visit	–	AF progression [i.e., paroxysmal to persistent or permanent, persistent to permanent] (Ref: paroxysmal or persistent AF)	Median (IQR), 2.78 (1.95–3) y	Model 1 uOR (CI), 1.05 (0.93–1.17)
								Age, sex, race, medical history, cardiovascular history			Model 2 aOR (CI), 0.96 (0.85, 1.08)
14	Schnabel, 2018	7 European countries	2151	Paroxysmal or persistent AF	72	39.9	NR	–	AF progression [i.e., paroxysmal to persistent or permanent, persistent to permanent] (Ref: paroxysmal or persistent AF)	Mean, 1 y	uOR (CI), 1.22 (0.98–1.52)
								Age, sex, country			aOR (CI), 1.23 (0.98–1.53)
								AF duration, HF, hyperthyroidism, no sinus rhythm, cardioversion, valvular heart disease			**aOR (CI), 1.29 (1.01–1.65)**

*DM* diabetes mellitus, *HR* hazard ratio, *AF* atrial fibrillation, *BMI* body mass index, *HTN* hypertension, *aHR* adjusted hazard ratio, *CI* 95% confidence interval, *uHR* unadjusted hazard ratio, *PAF* paroxysmal atrial fibrillation, *aOR* adjusted odds ratio, *uOR* unadjusted odds ratio, *NR* not reported, *CHD* coronary heart disease, *LVH* left ventricular hypertrophy, *HF* heart failure, *SBP* systolic blood pressure, *DBP* diastolic blood pressure, *CHD* coronary heart disease, *MI* myocardial infarction, *y* years, *IQR* interquartile range, *Ref* reference, *sd* standard deviation

*The studies investigated the association of diabetes with nonparoxysmal AF (compared to paroxysmal AF)

**Median follow-up time of the entire cohort of 34,720 women without AF at baseline, of which 1039 developed AF over follow-up

***HRs of developing non-paroxysmal AF compared to paroxysmal AF were calculated based on: (i) HR of developing paroxysmal AF compared to no AF; (ii) HR of developing non-paroxysmal AF compared to no AF; (iii) p-value from likelihood ratio tests of the null hypothesis that diabetes has an equal effect on the development of paroxysmal vs non-paroxysmal AF

^†^Odds ratios for developing non-paroxysmal AF compared to paroxysmal AF were calculated manually

### Cross-sectional association of diabetes with AF types

**Fig. 1:**
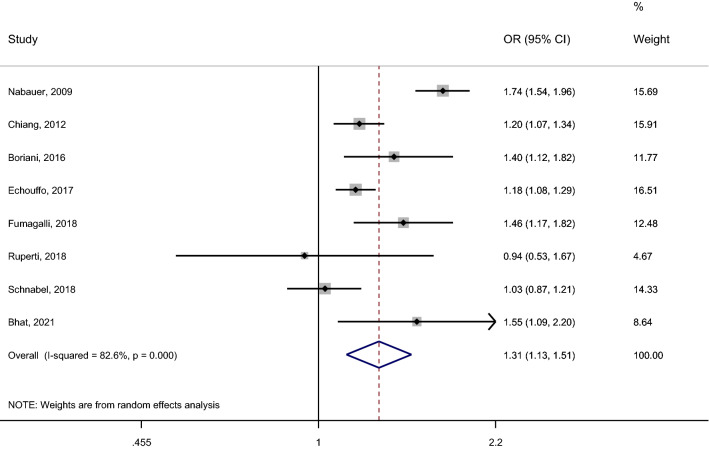
Cross- sectional association of diabetes with non-paroxysmal AF (vs paroxysmal AF). Studies were included in the meta-analysis if they assessed the crosss-sectional association of diabetes with the likelihood of having non-paroxysmal AF (vs paroxysmal AF) among patients with AF; and provided poolable estimates. *AF* atrial fibrillation, *OR* odds ratio, *95% CI* 95% confidence interval, *IV* inverse variance method, *I-squared* test for heterogeneity

Our systematic review included 8 studies performed among patients with AF investigating the cross-sectional association of diabetes with the likelihood of having non-paroxysmal AF rather than paroxysmal AF [13, 15, 16, 23, 27, 28, 34, 36] (Table 1). Of the eight studies, six studies reported unadjusted estimates [13, 15, 23, 27, 28, 36], and two studies reported adjusted and unadjusted estimates [16, 34] (Table 1). Of the eight studies, six studies showed an association of diabetes with an increased likelihood of non-paroxysmal AF (with ORs varying from 1.2 to 1.8) [13, 23, 27, 28, 34, 36]; and 2 studies [15, 16] showed no association. We included these 8 studies in the meta-analysis since all eight studies classified AF types into paroxysmal (reference) vs non-paroxysmal. The meta-analysis showed that diabetes is associated with a 1.31-times increased likelihood of having non-paroxysmal AF rather than paroxysmal AF with high heterogeneity (pooled OR [95% CI], 1.31 [1.13–1.51], I^2^ = 82.6%) (Fig. 1). All studies we included in our meta-analysis provided unadjusted estimates. Results remained consistent when pooling the two studies reporting adjusted estimates (including adjustments for age, sex, smoking, physical activity, cardiovascular risk factors, and cardiovascular diseases) with the six studies reporting unadjusted estimates (pooled OR [95% CI], 1.29 [1.11–1.50], I^2^ = 82.5%).

### Longitudinal association of diabetes with AF types

**Fig. 2:**
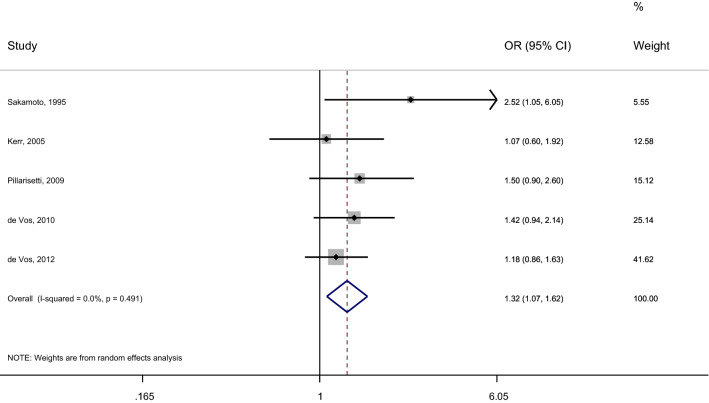
Longitudinal association of diabetes with non-paroxysmal AF (vs paroxysmal AF). Studies were included in the meta-analysis if they assessed the longitudinal association of diabetes with the likelihood of developing non-paroxysmal AF (vs paroxysmal AF) among patients with paroxysmal AF; and provided poolable estimates. *AF* atrial fibrillation, *OR* odds ratio, *95% CI* 95% confidence interval, *IV* inverse variance method, *I-squared* test for heterogeneity

Our systematic review included 14 studies investigating the longitudinal association of diabetes with AF types [7, 12, 14, 15, 23–26, 29–33, 35] (Table 2). Of the 14 studies, six studies reported unadjusted estimates [7, 26, 31–33, 35], six studies reported adjusted estimates [12, 14, 24, 25, 29, 30], and 2 studies reported adjusted and unadjusted estimates [15, 23] (Table 2). One study was performed among patients without AF at baseline; it showed no association between diabetes and the likelihood of developing non-paroxysmal AF rather than paroxysmal AF [25]. Five studies were performed among patients with paroxysmal or persistent AF at baseline; they showed no association between diabetes and the likelihood of developing permanent AF [24, 30] or “AF progression” (i.e., paroxysmal to persistent or permanent; persistent to permanent) over the follow-up period [15, 23, 29] (Table 2, Additional file 1: Table S3). Eight studies included patients with paroxysmal AF at baseline; these studies investigated the association of diabetes with the likelihood of developing either “permanent AF” [12, 14], “recurrent AF” [31], “chronic AF,” or “non-paroxysmal AF” [7, 26, 32, 33, 35] (Table 2, Additional file 1: Table S3). Of these 8 studies, 2 studies showed an association between diabetes and incident non-paroxysmal AF and permanent AF, respectively (Table 2) [14, 32]. The other 6 studies reported no association between diabetes and AF types (Table 2). We included 5 of 14 longitudinal studies in our meta-analysis; these 5 studies classified AF types into paroxysmal (reference) vs non-paroxysmal AF (Fig. 2). The meta-analysis showed that among patients with paroxysmal AF, diabetes was associated with a 1.32-times increased likelihood of developing non-paroxysmal AF with no heterogeneity (pooled OR [95% CI]; 1.32 [1.07–1.62]; I^2^ = 0%) (Fig. 2). All studies we included in our meta-analysis provided unadjusted estimates (Table 2, Fig. 2).

### Sensitivity analyses

Results from our meta-analyses of cross-sectional and longitudinal studies did not change substantially after removing studies one by one from the analyses (Figs. 1, 2, Additional file 1: Table S5). In the meta-analysis of cross-sectional studies, the heterogeneity decreased from high (I^2^, 82.6%) to moderate (I^2^, 44%) after removing the study from Nabauer et al. [13] (Additional file 1: Table S5). In the meta-analysis of longitudinal studies, results remained consistent after performing subgroup analyses based on follow-up time and also after restricting the analysis to studies that defined AF types in accordance with guidelines (Additional file 1: Table S6).

### Quality assessment

Of the 20 included studies, 12 (60%) were poor quality, 1 (5%) fair quality, and 7 (35%) good quality. Out of 9 stars, 6 studies scored 8 stars; 6 scored 6; 4 scored 5; and 4 scored 4. We present study quality assessment scores in Additional file 1: Table S7.

### Assessment of publication bias

We show funnel plots for the cross-sectional and longitudinal association of diabetes with non-paroxysmal AF (vs paroxysmal AF) in Additional file 1: Fig. S2. The Egger`s regression test did not show significant funnel plot asymmetry (p-values 0.8 and 0.2, respectively). Hence, even though it should be noted that a maximum of eight studies were included, there was no evidence of publication bias.

## Discussion

### Main findings

To the best of our knowledge, ours is the first systematic review which summarizes the literature regarding the association of diabetes with AF types in humans. Furthermore, this is the first meta-analysis which evaluates the likelihood of having non-paroxysmal AF rather than paroxysmal AF among patients with and without diabetes. We included various studies with relatively large sample sizes of participants with a wide range of ages from different parts of the world. The quality of studies spanned poor (60%), fair (5%), and good (35%). The meta-analysis of cross-sectional studies suggested that patients with AF and diabetes have a 1.31-times higher likelihood of non-paroxysmal AF when compared to those without diabetes. Our meta-analysis of longitudinal studies suggested that among patients with paroxysmal AF, the presence of diabetes is associated with 1.32-times higher likelihood of progression to non-paroxysmal AF. Additional sensitivity analyses provided similar findings. No evidence of publication bias was observed in included studies. The meta-analysis of cross-sectional studies indicated a high heterogeneity. However, the heterogeneity was reduced (I^2^ = 44%) in the “leave one out analysis” when removing the study from Nabauer et al. [13]. The study from Nabauer et al. was conducted over the time period 2004–2006, and it was the earliest investigation reporting on the cross-sectional association of diabetes with AF types, compared to the other studies. Hence, the result of the “leave-one out analysis” might be explained by the differences in the recruitment periods across studies, which can reflect differences over time in the definitions of AF types and management of patients with AF. No heterogeneity was observed in the meta-analysis of longitudinal studies.

### Mechanisms linking diabetes to non-paroxysmal AF

Several mechanisms linking diabetes to non-paroxysmal AF can be proposed, including progression of atrial remodelling, worsening atrial cardiomyopathy, and development of cardiometabolic diseases [6]. Diabetes can lead to left ventricular hypertrophy and dysfunction, which in turn increase atrial afterload and promote left atrial dilation. These conditions further foster the maintenance and recurrence of AF [26, 38]. Furthermore, diabetes is accompanied by an inflammatory state and oxidative stress, which are characterized by elevated levels of C-reactive protein, tumor necrosis factor-alpha, interleukin-6, and reactive oxygen species [9, 39]. Inflammation and oxidative stress are key mediators of proarrhythmic atrial remodeling among patients with diabetes [9, 39]. Diabetes is also associated with atrial fibrosis, which can promote gene expression that enhances the proliferation of fibroblasts and increases extra-cellular matrix secreting function [40]. This underlies the progression of AF to sustained forms by creating a long-term positive feedback loop—the so-called “AF begets AF” hypothesis [40]. Lastly, diabetes increases the risk of cardiometabolic diseases, such as coronary artery disease, hypertension, heart failure, and chronic kidney disease, which further increase susceptibility to non-paroxysmal AF [7, 29, 40–42].

### Lessons learned and future perspectives

Our study highlights the need for improving the quality of research about diabetes and AF types, while also proposing new research directions for the future. First, the studies we included in our systematic review used different categorizations of AF types, such as “paroxysmal” vs “non-paroxysmal” AF or “non-permanent” (i.e., paroxysmal or persistent) vs “permanent” AF. Although classification as paroxysmal or non-paroxysmal reflects the pathophysiology of AF, classification as non-permanent or permanent mainly reflects the therapeutic attitude of the patient and physician. For instance, older patients and those who have comorbidities are more likely to be considered as having permanent AF. Future studies aiming to provide mechanistic insights about diabetes and AF types should thus focus on the classification of AF types as paroxysmal and non-paroxysmal AF [6].

Second, longitudinal studies included in our systematic review focused on several transitions of AF types. One study followed-up patients without AF and classified them based on AF development (i.e., no AF, paroxysmal AF, non-paroxysmal AF) [25]. Some studies followed-up patients with paroxysmal AF and classified them based on recurrence [12, 14, 31]; and other studies followed-up patients with paroxysmal or persistent AF and classified them based on AF progression (i.e., paroxysmal to persistent or permanent; persistent to permanent) [15, 23, 29]. In general, AF was documented by medical records, clinical visits, ECG recordings and/or Holter monitoring, which were performed at baseline and over a limited number of follow-up visits. Indeed, AF is a dynamic disease. Over time, patients without AF can progress to paroxysmal or non-paroxysmal AF; and patients with paroxysmal AF can also progress to non-paroxysmal AF [33, 43]. Since evaluation requires a patient’s repeated long-term rhythm monitoring, evaluating AF types and progression can thus be challenging. In particular, the limited number and small frequency of AF evaluations may impede the detection of paroxysmal AF. This may further hamper the comparison between AF types. Future studies with long-term follow-up need to comprehensively account for the progressive nature of AF and evaluate the role of diabetes in these dynamic processes.

Third, all studies in our systematic review that investigated the longitudinal association of diabetes with AF progression only evaluated diabetes status at baseline. Future studies should account for the potential development of incident diabetes over time and also apply Cox models with time-varying covariates.

Fourth, most studies included in our systematic review did not describe how they defined diabetes. Some studies also used outdated terminology for AF types (e.g., “chronic AF”) [32, 33]. Future investigations thus need to define diabetes diagnosis in accordance with recommendations and use the most recent terminology for and definitions of AF types [6, 44].

Fifth, we observed different levels of adjustments across studies in the systematic review; some studies did not adjust for potential confounders, some adjusted for age and sex, and others adjusted for cardiovascular risk factors and/or cardiometabolic diseases. In general, effect estimates attenuated after adjusting for cardiovascular risk factors (e.g., body mass index, hypertension, systolic and diastolic blood pressure) and/or cardiometabolic diseases (e.g., coronary heart disease, revascularization, valvular heart disease, heart failure, stroke, renal failure, and thyroid dysfunction). This can be explained by the fact that some of these factors can be on the path linking diabetes to AF types. Future studies are warranted to clarify whether these factors have a mediating or confounding role in the association of diabetes with AF types. The studies that were eligible to be included in the meta-analyses of cross-sectional and longitudinal studies were mostly or completely unadjusted for potential confounders. In particular, adjusting for age and sex is crucial when investigating the association of diabetes with AF types, and future studies need to account for these factors. Additional adjustments for confounders, including alcohol, smoking, physical activity, education, medications, need to be performed accordingly.

Sixth, most studies included in the systematic review and meta-analysis did not take into account AF duration, which represents an important aspect in the examination of AF pattern. Hypothetically, underlying cardiovascular risk factors, such as diabetes, can be associated with a longer duration of atrial remodelling. In turn, AF duration can be associated with an increased likelihood of non-paroxysmal AF [45]. However, in one of the studies of our systematic review, the association between diabetes and non-paroxysmal AF became stronger and statistically significant after adjustment for AF duration, among other factors [15]. Future studies are needed to provide well characterized data on AF duration.

Although the results of our cross-sectional and longitudinal analyses were consistent, we can not exclude the possibility of residual or unmeasured confounding, due to the observational character of our study. Future studies (e.g., Mendelian Randomization) need to establish if the association of diabetes with non-paroxysmal AF is causal.

Finally, the increased risk of bias in our systematic review and meta-analysis was mainly explained by concerns related to definitions of AF types and adjustments for potential confounders. Our study highlights the need for improving these aspects and the overall quality of evidence in the future.

### Areas of limited evidence and future perspectives

*Diabetes and non-paroxysmal AF* We were able to examine two specific research questions in our meta-analyses: (1) “Among patients with AF, is the presence of diabetes associated with an increased likelihood of having non-paroxysmal AF rather than paroxysmal AF?” (2) and “Among patients with paroxysmal AF, is the presence of diabetes associated with an increased likelihood of progression to non-paroxysmal AF?” Another important research question would be: “Among patients without AF, is the presence of diabetes associated with an increased risk of developing non-paroxysmal AF rather than paroxysmal AF?” Since only one study investigated this research question, we could not perform a meta-analysis on this matter [25]. Future studies are needed to evaluate the association of diabetes with paroxysmal AF (vs no AF) and of diabetes with non-paroxysmal AF (vs no AF), and assess whether there are significant differences.

*Glycemic control and non-paroxysmal AF* Given the scarcity of evidence in the field, we could not perform a systematic review on the association between glycemic control and AF types. So far, increasing concentrations of glycated hemoglobin (HbA1c) and long term glycemic variability have been prospectively associated with an increased risk of incident AF [46–48]. In accordance, we hypothesize that patients with diabetes and inadequate glycemic control are more likely to have non-paroxysmal AF rather than paroxysmal AF. This hypothesis was also confirmed in a study reporting a positive linear association of HbA1c levels with non-paroxysmal AF [25]. Future studies may need to evaluate whether it is useful to integrate diabetes and measures of glycemic control in risk scores for predicting the risk of incident non-paroxysmal AF among those without AF or with paroxysmal AF, respectively.

*SGLT2 inhibitors and non-paroxysmal AF* Recent studies have shown that sodium dependent glucose cotransporter 2 inhibitors have beneficial effects reducing the risk of incident AF and improving AF prognosis [49–52]. It can thus be assumed that this therapeutic option could also reduce the risk of non-paroxysmal AF. However, further studies are warranted to confirm this assumption.

## Conclusions and implications

Our meta-analysis findings suggest that diabetes is associated with increased likelihood of non-paroxysmal AF rather than paroxysmal AF. Our systematic review provides a comprehensive summary of evidence about the association of diabetes with AF types. These insights allowed us to identify current limitations and propose new directions for the improvement of future research about diabetes and AF types. Specifically, future studies should be based on classifying AF types into paroxysmal vs non-paroxysmal AF, properly adjusting for confounders, accounting for incident diabetes using Cox models with time-varying covariates, as well as using standard definitions for diabetes and AF types in accordance with existing recommendations. Further high quality studies are needed to replicate our findings, examine causality, elucidate the exact mechanisms linking diabetes to non-paroxysmal AF, evaluate the potential value of diabetes in predicting non-paroxysmal AF, and assess the role of glycemic control and antidiabetic medications on AF types.

Our systematic review and meta-analysis provides insights into the pathophysiology of AF. Our findings suggest that patients with diabetes might need more attentive care than those without diabetes, in order to halt progression of AF burden and prevent adverse cardiovascular events. Our data can also imply that the substrate to ablate might be more complex in patients with diabetes than in those without diabetes. Future strategies can foster prevention of non-paroxysmal AF by close monitoring of patients at high risk, increasing patient awareness, and involving patients in treatment plans. In addition, future studies could explore markers that may be used in the clinical setting to identify patients with diabetes at increased risk of AF progression, thus improving personalized care.

## Supplementary Information

**Additional file 1.** Supplemental Material. **Appendix A.** Supplemental information on search strategy. **Appendix B.** Adapted scale from the Newcastle-Ottawa quality assessment scale for cohort studies. **Fig. S1.** Flowchart for study inclusion. **Fig. S2.** Funnel plots on the cross-sectional and longitudinal association of diabetes with non-paroxysmal AF (vs paroxysmal AF). **Table S1.** Classification of AF types, based on the presentation, duration, and spontaneous termination of AF episodes. **Table S2.** Recruitment setting. **Table S3.** AF definitions across studies. **Table S4.** AF monitoring in studies investigating the longitudinal association of diabetes with AF types. **Table S5.** "Leave one out" sensitivity analysis. **Table S6.** Sensitivity analyses in the meta-analysis of longitudinal studies investigating the association of diabetes with non-paroxysmal AF (vs paroxysmal AF). **Table S7.** Quality assessment scale.

## Data Availability

Data sharing is not applicable to this article as no datasets were generated or analyzed during the current study.

## Abbreviations

AF: Atrial fibrillation
HbA1c: Glycated hemoglobin
HR: Hazard ratio
OR: Odds ratio
95% CI: 95% Confidence intervals
NOS: Newcastle–Ottawa Scale
