# Supplementary material for: Association of diabetes with atrial fibrillation types: a systematic review and meta-analysis
Source: Cardiovasc Diabetol. 2021 Dec 7;20:230. doi: 10.1186/s12933-021-01423-2 (PMC8653594; doi:10.1186/s12933-021-01423-2)
Supplement: Supplementary file 1 — Additional file 1. Supplemental Material. Appendix A. Supplemental information on search strategy. Appendix B. Adapted scale from the Newcastle-Ottawa quality assessment scale for cohort studies. Fig. S1. Flowchart for study inclusion. Fig. S2. Funnel plots on the cross-sectional and longitudinal association of diabetes with non-paroxysmal AF (vs paroxysmal AF). Table S1. Classification of AF types, based on the presentation, duration, and spontaneous termination of AF episodes. Table S2. Recruitment setting. Table S3. AF definitions across studies. Table S4. AF monitoring in studies investigating the longitudinal association of diabetes with AF types. Table S5. "Leave one out" sensitivity analysis. Table S6. Sensitivity analyses in the meta-analysis of longitudinal studies investigating the association of diabetes with non-paroxysmal AF (vs paroxysmal AF). Table S7. Quality assessment scale. [file 12933_2021_1423_MOESM1_ESM.docx]

**Appendix A. Supplemental information on search strategy**

**Embase.com (1490 results)**

| **1) Atrial fibrillation progression, from paroxysmal to non-paroxysmal** |
| --- |
| (('atrial fibrillation'/exp AND ('disease exacerbation'/de OR 'severity of illness index'/de OR 'disease course'/mj)) OR ((('atrial fibril*' OR 'atrium fibril*' OR 'auricular fibril*' OR 'artrial fibril*' OR 'artrium fibril*' OR AF) NEAR/5 (paroxysmal OR non-paroxysmal OR nonparoxysmal OR persistent OR permanent OR nonpermanent OR sustained OR progress* OR develop* OR severit*))):ab,ti) |
| **2) Diabetes, glucose level, hemogobin A1c, hyperglycemia** |
| ('diabetes mellitus'/exp OR 'glucose blood level'/exp OR 'impaired glucose tolerance'/de OR 'impaired fasting glucose'/de OR 'hyperglycemia'/de OR 'fasting glucose'/de OR 'glycosylated hemoglobin'/exp OR 'glycemic control'/de OR (diabet* OR ((glucose) NEAR/3 (level* OR blood OR serum OR plasma* OR concentration* OR tolerance OR intolerance OR sensitiv* OR insensitiv* OR resistan* OR response OR dependen* OR homeosta*)) OR hypoglycemi* OR hypoglycaemi* OR hyperglycemi* OR hyperglycaemi* OR (fasting NEAR/2 glucose) OR 'glycated hemoglobin' OR 'glycated haemoglobin' OR 'glycosylated haemoglobin' OR 'glycosylated hemoglobin' OR HbA1c OR 'Hb A1c' OR 'hemoglobin A1c' OR 'haemoglobin A1c'):ab,ti) |
| **3) Observational studies** |
| ('cohort analysis'/exp OR 'longitudinal study'/exp OR 'prospective study'/exp OR 'follow up'/exp OR 'retrospective study'/de OR 'cross-sectional study'/de OR 'observational study'/de OR 'population research'/de OR 'case control study'/exp OR 'case study'/exp OR 'major clinical study'/de OR (cohort* OR ((prospectiv* OR populat* OR observ* OR retrospect* OR epidemiologic*) NEAR/3 (stud* OR trial*)) OR (case* NEAR/3 control*) OR (case* NEAR/3 series) OR (Cross NEAR/1 section*) OR 'case cohort*' OR 'nested case control*' OR prospectiv* OR longitudinal* OR 'follow up' OR followup OR population‐based):ab,ti) |

**Limits:** NOT ([animals]/lim NOT [humans]/lim) NOT ([Conference Abstract]/lim OR [Letter]/lim OR [Note]/lim OR [Editorial]/lim)

**Medline (Ovid) (888 results)**

| **1) Atrial fibrillation progression, from paroxysmal to non-paroxysmal** |
| --- |
| ((atrial fibrillation/ AND (disease progression/ OR "Severity of Illness Index"/ OR recurrence/)) OR (((atrial fibril* OR atrium fibril* OR auricular fibril* OR artrial fibril* OR artrium fibril* OR AF) ADJ5 (paroxysmal OR non-paroxysmal OR nonparoxysmal OR persistent OR permanent OR nonpermanent OR sustained OR progress* OR develop* OR severit*))).ab,ti.) |
| **2) Diabetes, glucose level, hemogobin A1c, hyperglycemia** |
| (exp diabetes mellitus/ OR blood glucose/ OR exp hyperglycemia/ OR Glycated Hemoglobin A/ OR (diabet* OR ((glucose) ADJ3 (level* OR blood OR serum OR plasma* OR concentration* OR tolerance OR intolerance OR sensitiv* OR insensitiv* OR resistan* OR response OR dependen* OR homeosta*)) OR hypoglycemi* OR hypoglycaemi* OR hyperglycemi* OR hyperglycaemi* OR (fasting ADJ2 glucose) OR glycated hemoglobin OR glycated haemoglobin OR glycosylated haemoglobin OR glycosylated hemoglobin OR HbA1c OR Hb A1c OR hemoglobin A1c OR haemoglobin A1c).ab,ti.) |
| **3) Observational studies** |
| (exp cohort analysis/ OR cross-sectional studies/ OR observational study/ OR case control studies/ OR case reports/ OR (cohort* OR ((prospectiv* OR populat* OR observ* OR retrospect* OR epidemiologic*) ADJ3 (stud* OR trial*)) OR (case* ADJ3 control*) OR (case* ADJ3 series) OR (cross ADJ1 section*) OR case cohort* OR nested case-control* OR prospectiv* OR longitudinal* OR follow-up OR followup OR population-based).ab,ti.) |

NOT (exp animals/ NOT humans/) NOT (letter OR news OR comment OR editorial OR congresses OR abstracts).pt.

**Cochrane CENTRAL (111 results)**Cochrane Central Register of Controlled Trials

| **1) Atrial fibrillation progression, from paroxysmal to non-paroxysmal** |
| --- |
| ((((atrial-fibril* OR atrium-fibril* OR auricular-fibril* OR artrial-fibril* OR artrium-fibril* OR AF) NEAR/5 (paroxysmal OR non-paroxysmal OR nonparoxysmal OR persistent OR permanent OR nonpermanent OR sustained OR progress* OR develop* OR severit*))):ab,ti) |
| **2) Diabetes, glucose level, hemogobin A1c, hyperglycemia** |
| ((diabet* OR ((glucose) NEAR/3 (level* OR blood OR serum OR plasma* OR concentration* OR tolerance OR intolerance OR sensitiv* OR insensitiv* OR resistan* OR response OR dependen* OR homeosta*)) OR hypoglycemi* OR hypoglycaemi* OR hyperglycemi* OR hyperglycaemi* OR (fasting NEAR/2 glucose) OR "glycated hemoglobin" OR "glycated haemoglobin" OR "glycosylated haemoglobin" OR "glycosylated hemoglobin" OR HbA1c OR "Hb A1c" OR "hemoglobin A1c" OR "haemoglobin A1c"):ab,ti) |
| **3) Observational studies** |
| ((cohort* OR ((prospectiv* OR populat* OR observ* OR retrospect* OR epidemiologic*) NEAR/3 (stud* OR trial*)) OR (case* NEAR/3 control*) OR (case* NEAR/3 series) OR (Cross NEAR/1 section*) OR case-cohort* OR nested-case-control* OR prospectiv* OR longitudinal* OR follow-up OR followup OR population‐based):ab,ti) |

**Web of Science Core Collection (656 results)**

| **1) Atrial fibrillation progression, from paroxysmal to non-paroxysmal** |
| --- |
| TS=((((atrial-fibril* OR atrium-fibril* OR auricular-fibril* OR artrial-fibril* OR artrium-fibril* OR AF) NEAR/5 (paroxysmal OR non-paroxysmal OR nonparoxysmal OR persistent OR permanent OR nonpermanent OR sustained OR progress* OR develop* OR severit*)))) |
| **2) Diabetes, glucose level, hemogobin A1c, hyperglycemia** |
| TS=((diabet* OR ((glucose) NEAR/3 (level* OR blood OR serum OR plasma* OR concentration* OR tolerance OR intolerance OR sensitiv* OR insensitiv* OR resistan* OR response OR dependen* OR homeosta*)) OR hypoglycemi* OR hypoglycaemi* OR hyperglycemi* OR hyperglycaemi* OR (fasting NEAR/2 glucose) OR "glycated hemoglobin" OR "glycated haemoglobin" OR "glycosylated haemoglobin" OR "glycosylated hemoglobin" OR HbA1c OR "Hb A1c" OR "hemoglobin A1c" OR "haemoglobin A1c")) |
| **3) Observational studies** |
| TS=((cohort* OR ((prospectiv* OR populat* OR observ* OR retrospect* OR epidemiologic*) NEAR/3 (stud* OR trial*)) OR (case* NEAR/3 control*) OR (case* NEAR/3 series) OR (Cross NEAR/2 section*) OR case-cohort* OR nested-case-control* OR prospectiv* OR longitudinal* OR follow-up OR followup OR population‐based)) |

**Google Scholar (first 200) of 993’000 results**

"atrial|atrium|auricular fibrillation" paroxysmal|"non-paroxysmal"|nonparoxysmal|persistent|permanent|nonpermanent|sustained|progression|development|severity diabetes|"blood glucose"|"glucose * level"|"glycated hemoglobin"|HbA1c|hyperglycemia

**Appendix B. Adapted Scale from the Newcastle-Ottawa quality assessment scale for cohort studies**

***Selection (max 4 star)***

1. *Representativeness of the exposed cohort*
2. Truly representative of the average in the target population (all subjects or random sampling) *
3. Somewhat representative of the average in the target population (non-random sampling) *
4. Selected group of users
5. No description of the derivation of the cohort
6. *Sample size*
7. Justified and satisfactory*
8. Not satisfied
9. *Ascertainment of the exposure (risk factor)*
10. Secure record (e.g., medical records) *
11. Structured interview*
12. Written self-report
13. No description the measurement tool
14. *Non-respondents*
15. Comparability between respondents and non-respondents characteristics is established, and the response rate is satisfactory*
16. The response rate is unsatisfactory, or the comparability between respondents and non-respondents is unsatisfactory
17. No description of the response rate or the characteristics of the respondents and the non-respondents

***Comparability (max 2 stars****)*

1. *The subjects in the different outcome groups are comparable, based on the study design and analysis. Confounding factors are controlled.*
2. Study controls for the most important factors (age, sex) *
3. Study controls for additional relevant factors**
4. Inadequate degree of control

***Outcome (max 3 stars)***

1. *Assessment of the outcome*
2. Definition of AF is in line with the current guidelines **
3. Definition of AF is provided, but not in line with the current guidelines *
4. No definition of AF is provided
5. *Statistical test*
6. The statistical test used to analyze the data is clearly described and appropriate, and the measurement of the association is presented, including the probability level (p-value) *
7. The statistical test is not appropriate, not described or incomplete*.*

**Fig. S1. Flowchart for study inclusion**

Records identified through database searching (n, 3345)

Duplicates excluded (**n, 1348**)

Records after duplicates removed (**n, 1997**)

Records screened (**n, 1997**)

**20** unique studies included in the systematic review evaluating the association of diabetes with AF types

Records excluded based on title and abstract

(**n, 1964**)

Full text articles assessed for eligibility (**n, 33**)

Article included after screening of the reference lists of eligible studies (**n, 3**)

Records excluded **(n, 16)**

- Review study (n, 1)
- Full text unavailable (n, 7)
- Not relevant to the research question (n, 8)

**13** studies included in the meta-analysis evaluating the association of diabetes with non-paroxysmal AF

- **5** longitudinal studies
- **8** cross-sectional studies

**Fig. S2. Funnel plots on the cross-sectional and longitudinal association of diabetes with non-paroxysmal AF (vs paroxysmal AF).**

The log odds ratios are plotted against the standard error of the logarithm of the odds ratio. The dashed lines depict the logarithm of the summary odds ratio with its 95% confidence interval. (A) Cross-sectional association; (B) Longitudinal association. Abbreviation: OR, odds ratio.

**A.**

**B.**

| **Table S1. Classification of AF types, based on the presentation, duration, and spontaneous termination of AF episodes*** | |
| --- | --- |
| **AF type** | **Criteria** |
| First diagnosed AF | if not diagnosed before, irrespective of its duration or the presence/severity of AF-related symptoms |
| Paroxysmal AF | if it terminates spontaneously or with intervention within 7 days of onset |
| Persistent AF | if it is continuously sustained beyond 7 days, including episodes terminated by cardioversion (ie, drugs or electrical cardioversion) after ≥7 days |
| Long-standing persistent AF | if it is a continuous AF of >12 months’ duration when decided to adopt a rhythm control strategy |
| Permanent AF | if it is accepted by the patient and the physician, and no further attempts to restore/maintain sinus rhythm will be undertaken. Permanent AF represents a therapeutic attitude of the patient and the physician rather than an inherent pathophysiological attribute of AF |
| *Based on the recent guidelines from the European Heart Rhythm Association (EHRA) and European Society of Cardiology (Reference. Hindricks G, Potpara T, Dagres N, Arbelo E, Bax JJ, Blomström-Lundqvist C, et al. 2020 ESC Guidelines for the diagnosis and management of atrial fibrillation developed in collaboration with the European Association for Cardio-Thoracic Surgery (EACTS). Eur Heart J. 2021;42(5):373-498.)  Abbreviation: AF, atrial fibrillation. | |

| **Table S2. Recruitment setting** | | | | | |
| --- | --- | --- | --- | --- | --- |
|  | **Study** | **Setting** | **Design*** | **Recruitment period** | **Inclusion/Exclusion criteria** |
| **Studies investigating the cross-sectional association of diabetes with AF types** | | | | | |
| 1. | Nabauer, 2009 | Multicenter study enrolling patients from regional hospitals and practices of cardiologists, internists, and general practitioners, in Germany | Cross-sectional | 2004 to 2006 | Patients were included in the registry if they were 18 years or older  and had documented AF, either at the time of enrolment or during the preceding 12 months. Patients with atrial flutter as the sole arrhythmia were not included. |
| 2. | Chiang, 2012 | International survey at >800 sites in 26 countries | Cross-sectional | 2009 to 2010 | Patients with a history of AF, with at least 1 documented AF episode in the previous 12 months, or documented current AF, were enrolled. Exclusion criteria were: mental disability (eg, dementia or significant cognitive disorders), inability to pro­vide written informed consent, postoperative AF within 3 months of cardiac surgery, and participation in clinical trials investigating AF or antithrombotics during the previous month. |
| 3. | Boriani, 2016 | EURObservational Research Programme–AF General Pilot Registry, including inpatients and outpatients with AF, enrolled at 67 centres in 9 countries. | Cross-sectional | 2012 | Enrolment required documented AF, with a qualifying episode of AF documented in the 12 months prior to enrolment. |
| 4. | Echouffo-Tcheugui, 2017 | ORBIT-AF registry, an observational, prospective study. Patients were enrolled from heterogeneous practices across United States, including internal medicine, neurology, cardiology, and electrophysiology clinics. | Cross-sectional | 2010 to 2011 | The registry enrolled adults older than 18 years with documented AF. Patients were excluded if they were diagnosed as having AF secondary to an easily reversible condition, if they had a life expectancy <6 months, or if they did not have follow-up data on AF. |
| 5. | Fumagalli, 2018 | EORP-AF Registry investigated  how ESC participating countries manage patients with AF. 70 centres in 9 European Countries provided inpatient and outpatient data on AF. | Cross-sectional | 2012 to 2013 | Eligible patients had an AF episode which should have occurred in the last 12 months, independently of its presence at the time of enrollment. A primary or secondary diagnosis of AF was allowed. |
| 6. | Ruperti, 2018 | Basel atrial fibrillation cohort (BEAT-AF)  Study, a multicentre cohort study  among AF patients recruited from inpatient and outpatient clinics in Switzerland. | Cross-sectional | 2010 to 2014 | Consecutive patients with documented AF were included. The  enrolment of patients with acute illnesses was postponed until their health status had stabilised. Out of a population of patients with documented AF, only those with AF duration less than 2 years were included. |
| 7. | Schnabel, 2018 | 2 European registries on AF, (i) PREFER in AF (PREvention oF thromboembolic events-European Registry in Atrial Fibrillation) and (ii) its Prolongation study. | Cross-sectional | (i) PREFER in AF: 2012 to January 2014; (ii) The PREFER in AF | (i) PREFER in AF: Patients with AF at least 18 years; (ii) The PREFER in AF prolongation: Participants also had to be on non-vitamin K antagonist oral anticoagulant treatment at enrolment |
| 8. | Bhat, 2021 | Cardiology service in a hospital in Australia | Cross-sectional | 2013 to 2018 | Consecutive patients admitted with a primary diagnosis of AF were included. Patients with valvular AF, and patients without a comprehensive transthoracic echocardiogram were excluded. |
| **Studies investigating the longitudinal association of diabetes with AF types** | | | | | |
|  | Tsang 2007 | Community based cohort study in Olmsted County | Retrospective | 1980 to 2000 | Participants who had medical information in databases of Mayo clinic, with first diagnosed AF event of paroxysmal type, and who had follow-up data on AF types. |
|  | Pappone 2008 | Patients admitted to the emergency department | Prospective | January to July 2002 | Consecutive patients with first episode of paroxysmal AF were included. Patients with arrhythmia due to potentially reversible causes, such as acute or recent (<6 months) MI, recent cardiac surgery, NYHA class III–IV heart failure, severe valvular heart disease requiring surgery, uncontrolled hypertension, acute pulmonary disease, Wolff-Parkinson-White syndrome, a history of long QT syndrome, Brugada syndrome, pericarditis, substance abuse, electrolyte imbalance, hyperthyroidism, or hypothyroidism were excluded. Patients with hepatic disease, contraindication to anticoagulation therapy, or any condition making survival unlikely for 1 year were excluded. |
|  | Kawara 2010 | AF patients recruited through a retrospective questionnaire survey | Retrospective | May to November 2008 | Patients with AF |
|  | Thacker 2013 | “Group Health”, a population-based inception cohort study. | NS | 2001 to 2004 | Participants with newly diagnosed AF were included. Exclusion criteria were: (i) initial AF episode did not terminate within 6 months after onset, (ii) missing BMI values, (iii) BMI < 18.5 kg/m2 (underweight), (iv) less than 6 months of follow-up after initial AF episode terminated, (v) AF that occurred as part of a hospitalized terminal illness, (vi) peacemaker implanted before initial AF episode. |
|  | Senoo, 2014 | Hospital-based cohort of cardiovascular patients | Retrospective | 2004 to 2012 | The study included new patients visiting the Cardiovascular Institute Hospital in Tokyo, who had paroxysmal AF. Patients with active cancer and foreign travellers were excluded. |
|  | Sandhu 2014 | Women’s Health Study, a randomized placebo-controlled study on the role of aspirin, beta-carotene, and vitamin E in primary prevention of CVD and cancer | Prospective | 1993 to 2004 | Women who were 45 years or older, without known CVD, cancer, other major illnesses, and who had follow-up data available were included. |
|  | Blum 2019 | Basel-AF (BEAT-AF) and Swiss-AF, two multicenter cohorts of AF patients | Prospective | (i) BEAT-AF: 2010-2014; (ii) Swiss-AF: 2014-2017 | Patients with documented AF, who did not have secondary forms of AF (eg, AF after cardiac surgery) were included. Patients who had an acute illness within the past 4 weeks could only be enrolled once the acute episode was resolved. Patients with permanent AF at baseline, and those without follow-up data available were excluded. |
|  | Sakamoto, 1995 | Patients with AF who visited the Institute for Adult Diseases, Asahi Life Foundation, Tokyo | Retrospective | 1969 to 1990 | Patients with non-rheumathic AF of new onset were included. Patients treated with electrical cardioversion were excluded. |
|  | Kerr, 2005 | Canadian Registry of AF (CARAF), including patients at their first diagnosed AF, with data from family physicians, specialists, emergency rooms, and hospitalizations for other diagnoses. | NS | 1990 to 1996 | Patients with paroxysmal AF were included. Patients who had AF up to the 3 months visit without documentation of return to sinus rhythm were considered to have chronic AF and were excluded. Those who developed AF during hospitalization for heart surgery were excluded. |
|  | Pilarisetti, 2009 | Patients fol­lowed in the cardiology clinics at University of Kansas Hospitals | Retrospective | 1999 to 2007 | Patients with paroxysmal AF were included. Patients whose initial diagnosis was persistent or permanent AF were excluded. |
|  | De Vos 2010 | Euro Heart Survey on AF, enrolling consecutive patients at cardiology departments from 182 hospitals in 35 countries of the European Society of Cardiology | NS | 2003 to 2004 | The study included patients older than 18 years, with paroxysmal AF and first detected AF in whom sinus rhythm restored spontaneously or after pharmacological treatment during admission, and with 1 year follow-up. Patients who underwent electrical cardioversion to sinus rhythm were not included. |
|  | De Vos 2012 | RecordAF, an international longitudinal prospective cohort study in AF patients | Prospective | 2007 to 2008 | Patients were included when aged ≥18 years with AF discovered at the inclusion visit or AF diagnosed ≤1 year from inclusion. Exclusion criteria were: AF resulting from a transient cause, post-cardiac surgery AF, life expectancy of 1 year owing to a severe disease; mental disability; a pacemaker or an implantable cardioverter/defibrillator; scheduled pulmonary vein isolation, atrioventricular node/His bundle ablation, or pacemaker implantation; participation in an AF clinical trial in the previous 3 months; pregnancy or breastfeeding. |
|  | Echouffo-Tcheugui, 2017 | ORBIT-AF registry, an observational, prospective study. Patients were enrolled from heterogeneous practices across United States, including internal medicine, neurology, cardiology, and electrophysiology clinics. | Prospective | 2010 to 2011 | The registry enrolled adults older than 18 years with documented AF. Patients were excluded if they were diagnosed as having AF secondary to an easily reversible condition, if they had a life expectancy <6 months, or if they did not have follow-up data on AF. |
|  | Schnabel, 2018 | 2 European registries on AF, PREFER in AF (PREvention oF thromboembolic events-European Registry in Atrial Fibrillation) and its Prolongation study. | Prospective | (i) PREFER in AF: 2012 to January 2014; (ii) PREFER in AF prolongation: June 2014 to 2015 | (i) PREFER in AF included patients with AF at least 18 years; (ii) The PREFER in AF prolongation included patients who were on non-vitamin K antagonist oral anticoagulant treatment at enrolment |
| *Applicable to our specific research question  Abbreviations: AF, atrial fibrillation; BMI, body mass index; CVD, cardiovascular disease; ESC, European Society of Cardiology; MI, myocardial infarction; NYHA, New York Heart Association; NS, not specified. | | | | | |

| **Table S3. AF definitions across studies** | | | |
| --- | --- | --- | --- |
|  | **First author, year** | **AF definitions; (if applicable) illustration of AF transition** | |
| **Studies investigating the cross-sectional association of diabetes with AF types** | | | |
| 1 | Nabauer,  2009 | Paroxysmal AF was defined as AF that terminates spontaneously and generally lasts less than or equal to 7 days (usually <24 h). Persistent AF was defined as recurrent or sustained AF that does not terminate spontaneously and usually lasts more than 7 days; termination with pharmacological therapy or electrical cardioversion does not change the designation. Permanent AF was defined as long-standing AF in which cardioversion has failed or has not been attempted. | |
| 2 | Chiang,  2012 | Patients with paroxysmal, persistent and permanent AF were included. Definitions of AF types are not provided. | |
| 3 | Boriani,  2016 | Paroxysmal AF is self-terminating, usually within 48 hours. Although AF paroxysms may continue for up to 7 days, the 48 hours time point is clinically important; after this time the likelihood of spontaneous conversion is low and anticoagulation must be considered. Persistent AF is present when an AF episode either lasts longer than 7 days or requires termination by cardioversion, either with drugs or by direct current cardioversion. Long-standing persistent AF has lasted for ≥1 year when it is decided to adopt a rhythm control strategy. Permanent AF is present when the presence of the arrhythmia is accepted by the patient and physician. Patients with persistent AF and long-standing persistent AF were considered together. Patients presenting with persistent AF, but with history of paroxysmal AF, were classified as patients with persistent AF. | |
| 4 | Echouffo-Tcheugui, 2017 | Non-paroxysmal AF included persistent and permanent AF. Paroxysmal AF was defined as recurrent AF episodes that terminate spontaneously within 7 days; persistent AF as recurrent AF that is sustained for more than 7 days; and permanent AF as AF in which the presence of the AF is accepted. | |
| 5 | Fumagalli, 2018 | Non-paroxysmal AF included persistent, long-standing persistent, and permanent AF. Definitions of paroxysmal, persistent, and permanent AF were not provided. | |
| 6 | Ruperti.  2018 | Out of a population of patients with documented AF, only those with AF duration less than 2 years were included. Patients were then categorized into those with “recent onset paroxysmal AF” and “recent onset non-paroxysmal AF”. Paroxysmal AF was defined as AF that terminates spontaneously within 7 days of onset. Non-paroxysmal AF was defined as: (i) persistent AF, which was sustained for more than 7 days or required cardioversion; (ii) permanent AF, which was recorded if restoration of sinus rhythm was not possible or not further attempted. | |
| 7 | Schnabel,  2018 | The type of AF was classified according to European Guidelines. Paroxysmal AF is self-terminating, usually within 48 hours. Although paroxysmal AF may continue for up to 7 days, the 48 hours time point is clinically important because after this the likelihood of spontaneous conversion is low and anticoagulation must be considered. Persistent AF is present when an AF episode either lasts longer than 7 days or requires termination by cardioversion, either with drugs or by direct current cardioversion. Long-standing persistent AF has lasted for ≥1 year when it is decided to adopt a rhythm control strategy. Permanent AF is present when the presence of the arrhythmia is accepted by the patient and physician. | |
| 8 | Bhat, 2021 | Classification of AF was in accordance with the guidelines. Paroxysmal AF was defined as self-terminating AF within 7 days. Persistent AF was defined as lasting longer than 7 days with termination typically requiring pharmacological intervention or electrical cardioversion. Permanent AF was defined as AF where rhythm control strategies were no longer pursued or deemed futile. | |
| **Studies investigating the longitudinal association of diabetes with AF types** | | | |
| **A. Studies providing hazard ratios** | | | |
| 1 | Tsang, 2007 | Patients diagnosed with the first episode of paroxysmal AF were recruited. Participants were followed-up and were classified according to AF types. Paroxysmal AF was defined as recurrent, intermittent AF that was self-limiting and terminated without specific therapy. Persistent AF was defined as recurrent, sustained AF that was terminated by pharmacological or electrical intervention. Permanent AF was defined as continuous AF that could not be converted to normal sinus rhythm by pharmacological or electrical techniques. |  |
| 2 | Pappone,  2008 | Patients diagnosed with the first episode of paroxysmal AF were recruited. The “first episode of paroxysmal AF” was defined as the first recognized AF event for the individual without previous antiarrhythmic drug therapy. Participants were then followed-up. Over time, some of them had recurrent AF and others no AF recurrence. Paroxysmal AF was defined as recurrent AF that was self-terminating with episode durations less than 7 days. Persistent AF was defined as recurrent AF that was not self-terminating with episode duration more than 7 days. Permanent AF was defined as AF that had been present for at least 6 months without intervening spontaneous episodes of sinus rhythm for which cardioversion was unsuccessful and subsequently not attempted. |  |
| 3 | Kawara,  2010 | AF was classified into permanent and non-permanent. Permanent AF was defined as AF lasting >6 months. The rest of participants were classified as “non-permanent AF”. |  |
| 4 | Thacker,  2013 | Patients with newly diagnosed AF, whose initial AF terminated within 6 months and who had at least 6 months of subsequent follow-up were included. A minimum of 6 months was required to establish the occurrence of permanent AF. Therefore, people who had less than 6 months of follow-up after the initial AF episode terminated were excluded, because their follow-up time was insufficient to establish the occurrence of permanent AF. For the same reason, people who had sufficient follow-up time but did not meet the definition of permanent AF were censored 6 months prior to the end of follow-up. Eligible participants were followed-up for the development of recurrent AF. Recurrent AF was defined as documented AF episode after the initial AF episode. Permanent AF was defined as AF present on two separate occasions at least 6 months apart and no more than 36 months apart, without any documented sinus rhythm between the two occasions. Participants who met the definition of permanent AF were subset of those who had recurrent AF. |  |
| 5 | Senoo, 2014 | Patients with paroxysmal AF were recruited. At the initial visit, paroxysmal AF was defined as: (1) sinus rhythm on ECG and previous diagnosis of paroxysmal AF by referring physicians; (2) symptomatic AF on ECG at the initial visit and duration of AF estimated as <7 days according to symptoms or ECG recordings; and (3) asymptomatic AF on ECG at the initial visit and no AF 1 week prior. Patients whose AF was estimated to continue for >7 days after the initial visit, were considered to have persistent AF originally and were excluded from the analysis. At follow-up, the onset of recurrent AF was defined as the first time in which all ECGs indicated AF after ≥3 consecutive ECG at an interval ≥1 week after the initial examination. When an ECG could not be obtained thrice during the period, the physicians made a clinical judgment regarding the onset time of AF progression. When electrical cardioversion was performed after >7 days continuing from AF onset, it was also considered as AF progression. |  |
| 6 | Sandhu,  2014 | Patients without AF were followed-up and only those who developed paroxysmal and non-paroxysmal AF were selected. The null hypothesis was that diabetes had a similar effect on the development of paroxysmal and non-paroxysmal AF. Paroxysmal AF was defined as self-terminating within 7 days. Non-paroxysmal AF was defined as: (i) “persistent AF”, which required cardioversion or lasted ≥ 7 days; or “permanent AF”, which lasted > 1 year and/or attempts to convert rhythm were abandoned. |  |
| 7 | Blum, 2019 | At baseline, patients with paroxysmal and persistent AF were included and those with permanent AF were excluded. Paroxysmal AF was defined as self‐terminating, usually within 48 hours. Persistent AF was defined as episodes lasting >7 days or requiring termination by electrical or medical cardioversion. Permanent AF was defined as the patient and the physician accepting AF, and no further attempts to restore sinus rhythm are performed. AF progression was defined as AF progression from paroxysmal AF at baseline to non-paroxysmal AF (persistent or permanent AF) at the latest follow-up or as AF progression from persistent AF at baseline to permanent AF at the latest follow-up. |  |
| **B. Studies providing odds ratios** | | | |
| 8 | Sakamoto,1995 | Patients with «new onset of AF» (equivalent term used in the study for paroxysmal AF) were followed-up. Based on AF progression over follow-up, participants were categorized in paroxysmal and chronic AF. Paroxysmal AF consisted of patients in whom AF remained paroxysmal during the first follow-up year. Chronic AF consisted of patients in whom AF did not return to normal sinus rhythm for at least 6 months during first year of follow-up. |  |
| 9 | Kerr,  2005 | All patients were presumed to have paroxysmal AF at the time of diagnosis. Patients who had AF up to the 3 month visit without documentation of return to sinus rhythm were considered to have chronic AF and were excluded. Patients were followed-up. Recurrent paroxysmal AF was defined as an episode of AF by history or ECG documentation with subsequent demonstration of reversion to sinus rhythm. Chronic AF was defined as the ECG documentation of AF on 2 consecutive ECGs separated by a minimum of 1 week. For the purposes of the analysis, patients were classified as chronic AF patients at visit t if they were determined to be in chronic AF and were classified as having ongoing chronic AF for the next annual visit with no documentation of sinus rhythm in the interim. |  |
| 10 | Pillarisetti,  2009 | Patients with paroxysmal AF were included and those with persistent/permenent AF were excluded. Over follow-up, patients were divided in 2 groups: (i) those who remained in the group of paroxysmal AF, and (ii) those who progressed to persistent or permanent AF. AF types were defined based on ACC/AHA guidelines. Spontaneous conversion of AF to sinus rhythm or termination within 24 hours of onset was considered paroxysmal AF. If cardioversion was required or if AF persisted beyond 7 days, it was classified as persistent AF. AF refractory to cardioversion and long-lasting AF was classified as permanent AF. |  |
| 11 | de Vos, 2010 | The study included patients with paroxysmal AF and first detected AF in whom sinus rhythm restored spontaneously or after pharmacological treatment during admission. Patients who underwent electrical cardioversion to sinus rhythm were not included. Paroxysmal AF was defined as episodes of the arrhythmia that terminate spontaneously. Over follow-up, patients were categorized in 2 groups: (i) patients who remained in the category of paroxysmal AF; (ii) patients who progressed. AF progression was defined as paroxysmal AF at baseline becoming persistent or permanent AF at 1-year follow-up or first detected AF at baseline with spontaneous conversion to sinus rhythm during admission becoming persistent or permanent AF at 1 year follow-up. |  |
| 12 | de Vos, 2012 | Patients with paroxysmal AF and first-detected AF in whom sinus rhythm restored spontaneously or after pharmacologic treatment during admission were included. Over follow-up, patients were categorized in 2 groups: (i) patients who remained in the category of paroxysmal AF; (ii) patients who progressed. AF progression was defined as a change from self-terminating or paroxysmal AF at baseline to non–self terminating AF, that is persistent or permanent AF after 1 year of follow-up. AF type was defined based on the clinical judgement of the attending physician. |  |
| 13 | Echouffo-Tcheugui,  2017 | At baseline, patients with paroxysmal and persistent AF were included and those with permanent AF were excluded. Paroxysmal AF was defined as recurrent AF episodes that terminate spontaneously within 7 days; persistent AF as recurrent AF that is sustained for more than 7 days; and permanent AF as AF in which the presence of AF is accepted. AF progression was defined as: (i) progression from paroxysmal AF at baseline to either persistent or permanent AF reported at follow-up; or (ii) progression from persistent AF at baseline to permanent AF reported at any subsequent follow-up visit. |  |
| 14 | Schnabel,  2018 | Patients with paroxysmal and persistent AF were included. AF progression occurred if patients with paroxysmal AF at baseline were classified as persistent or permanent AF at follow-up and if patients with persistent AF on enrollment were diagnosed with permanent AF after one year. As there was a relatively small number of patients with longstanding persistent AF, this category was categorized with permanent AF for analysis. AF types were defined in accordance with the European Society of Cardiology (ESC) and European Society of Cardiothoracic surgery (EATCS) guidelines. No specific description of AF types was provided. |  |
| Abbreviations: AF, atrial fibrillation; DM, diabetes mellitus; ECG, electrocardiogram. | | | |

| **Table S4. AF monitoring in studies investigating the longitudinal association of diabetes with AF types** | | |
| --- | --- | --- |
| **Studies** | **AF evaluation** | **Time points of AF monitoring** |
| Tsang, 2007 | ECG, clinical documentation | Baseline, 3 years |
| Pappone, 2008 | ECG and 48-hour Holter for AF monitoring.  In addition, patients were provided with a LifeWatch event monitor  and were asked to record their rhythm at least 5 days per week for 3 minutes and whenever they had symptoms suggestive of AF. | Baseline, 1 month, 3 months, 6 months, and annually thereafter for 5 years |
| Kawara, 2010 | Consecutive ECG recordings and medical charts | NR |
| Thacker, 2013 | Rhythm documentation obtained from: 1) medical records (including ECGs, Holter monitors, rhythm strips, and electrical cardioversion), 2) Group Health ECG database (including dates and results of ECGs), and 3) Group Health administrative databases (including dates of electrical cardioversion, AF ablation, and maze procedures). | Baseline, follow-ups* |
| Sandhu, 2014 | Medical records, ECGs, rhythm strips, and 24-hour ECG monitoring | Baseline, 2 years, annually thereafter |
| Senoo, 2014 | ECGs, clinical examinations | Baseline, ≥3 examinations at an interval ≥1 week after the initial examination |
| Blum, 2019 | Medical reports, clinical visits (Swiss-AF) or telephone interviews (BEAT-AF). | Baseline, then yearly follow-ups |
| Sakamoto, 1995 | Routine 12-lead ECGs | Baseline, several follow-up examinations (ECGs were recorded 7.7 times over the first year) |
| Kerr, 2005 | ECG | Baseline, then yearly follow-ups |
| Pillarisetti, 2009 | Medical records | Several clinical encounters |
| de Vos, 2010 | Medical records and medical information systems | Baseline, 1 year |
| de Vos, 2012 | Routine follow-up visits | Baseline, 6 months, 1 year |
| Echouffo-Tcheugui, 2017 | ECG, medical records | Baseline, 6 months, 1 year, 1 year and half, 2 years, 2 years and half, 3 years |
| Schnabel, 2018 | ECG, medical records | Baseline, 1 year |
| Abbreviations: AF, atrial fibrillation; ECG, electrocardiogram; NR, not reported  * The medical record review took place a mean of 2 years after the initial AF episode, with a range of 6 months to 5 years. Data from the ECG database and administrative databases were available for a mean of 7 years after the initial AF episode, with a range of 5 to 8 years. | | |

| **Table S5. “Leave one out” sensitivity analysis*** | | |
| --- | --- | --- |
|  | **Name of study removed** | **OR (95% CI); I^2^ [%]** |
| ***A*** | ***Cross- sectional association of diabetes with non-paroxysmal AF***  Pooled estimate in the main analysis: OR (95% CI), 1.31 (1.13 -1.51); I^2^ = 82.6% | |
|  | Nabauer, 2009 | 1.22 (1.11-1.34); 44% |
|  | Chiang, 2012 | 1.33 (1.11-1.59); 84% |
|  | Boriani, 2016 | 1.30 (1.11-1.52); 84% |
|  | Echouffo, 2017 | 1.33 (1.12-1.59); 82% |
|  | Fumagalli, 2018 | 1.29 (1.10-1.51); 84% |
|  | Ruperti, 2018 | 1.33 (1.15-1.54); 84% |
|  | Schnabel, 2018 | 1.36 (1.17-1.59); 81% |
|  | Bhat, 2021 | 1.29 (1.10-1.50); 84% |
|  | | |
| ***B*** | ***Longitudinal association of diabetes with non-paroxysmal AF***  Pooled estimate in the main analysis: OR (95% CI), 1.32 (1.07 -1.62); I^2^ = 0% | |
|  | Sakamoto, 1995 | 1.27 (1.03-1.57); 0% |
|  | Kerr, 2005 | 1.36 (1.09-1.70); 0% |
|  | Pillarisetti, 2009 | 1.30 (1.03-1.63); 4% |
|  | de Vos, 2010 | 1.30 (1.01-1.68); 7% |
|  | De Vos, 2012 | 1.43 (1.09-1.87); 0% |
| *To evaluate the role of individual studies on the overall results, we recalculated the effect estimates after removing the studies one by one from the pooled analyses. Effect estimates on the association of diabetes with non-paroxysmal AF were pooled using random effects models.  Abbreviations: AF, atrial fibrillation; OR, odds ratio; 95% CI, 95% confidence interval; I^2^, heterogeneity. | | |

| **Table S6. Sensitivity analyses in the meta-analysis of longitudinal studies investigating the association of diabetes with non-paroxysmal AF (vs paroxysmal AF)*** |
| --- |
| 1. ***Subgroup analysis based on follow-up time*** |
| Follow-up time **≤**1 year: 3 studies; OR (95% CI), 1.37 (1.02-1.86); I^2^, 25% |
| Follow-up time > 1 year: 2 studies; OR (95% CI), 1.29 (0.87-1.90), I^2^, 0% |
| 1. ***Sensitivity analysis restricted to studies that defined AF types in accordance with recommendations*** |
| 3 studies; OR (95% CI), 1.31 (1.04-1.64), I^2^, 0% |
| *We performed subgroup analyses to evaluate the influence of follow-up time on our results. To evaluate whether the definition of AF types would have an influence on our results, we restricted the analysis to studies that defined AF types in accordance with recommendations. Effect estimates on the association of diabetes with non-paroxysmal AF were pooled using random effects models.  Abbreviations: AF, atrial fibrillation; OR, odds ratio; 95% CI, 95% confidence interval; I^2^, heterogeneity. |

| **Table S7. Quality Assessment Scale** | | | | | |
| --- | --- | --- | --- | --- | --- |
| **First author, year (Reference)** | **Selection (maximum 4 stars)** | **Comparability (maximum 2 stars)** | **Outcome (maximum 3 stars)** | **Total number of stars** | **Quality rating** |
| Sakamoto, 1995 | *** |  | ** | 5 | Poor quality |
| Kerr, 2005 | *** |  | ** | 5 | Poor quality |
| Tsang, 2007 | *** | * | ** | 6 | Good quality |
| Pappone, 2008 | *** | ** | *** | 8 | Good quality |
| Nabauer, 2009 | *** |  | * | 4 | Poor quality |
| Pillarisetti, 2009 | *** |  | *** | 6 | Poor quality |
| Kawara, 2010 | ** | ** | ** | 6 | Fair quality |
| de Vos, 2010 | *** |  | ** | 5 | Poor quality |
| Chiang, 2012 | *** |  | * | 4 | Poor quality |
| de Vos, 2012 | *** |  | ** | 5 | Poor quality |
| Thacker, 2013 | *** | ** | * | 6 | Poor quality |
| Senoo, 2014 | *** |  | *** | 6 | Poor quality |
| Sandhu, 2014 | *** | ** | *** | 8 | Good quality |
| Boriani, 2016 | *** |  | * | 4 | Poor quality |
| Echouffo-Tcheugui, 2017 | *** | ** | *** | 8 | Good quality |
| Schnabel, 2018 | *** | ** | * | 6 | Poor quality |
| Fumagalli, 2018 | *** |  | * | 4 | Poor quality |
| Ruperti, 2018 | *** | ** | *** | 8 | Good quality |
| Blum, 2019 | *** | ** | *** | 8 | Good quality |
| Bhat, 2021 | *** | ** | *** | 8 | Good quality |
| *The thresholds for converting the NOS scores into the Agency for Healthcare Research and Quality (AHRQ) standards, the quality of the studies were categorized as follows: (I) Good quality: 3 or 4 stars in the selection domain, AND 1 or 2 stars in the comparability domain, AND 2 or 3 stars in the outcome domain. (II) Fair quality: 2 stars in the selection domain, AND 1 or 2 stars in the comparability domain, AND 2 or 3 stars in the outcome domain. (III) Poor quality: 0 or 1 star in the selection domain, OR 0 star in the comparability domain, OR 0 or 1 stars in the outcome domain. | | | | | |
